# Severe hypotension and postoperative cardiac arrest caused by 5-aminolevulinic acid: a case report

**DOI:** 10.1186/s13256-024-04589-x

**Published:** 2024-05-30

**Authors:** Taishi Miyazaki, Shinya Taguchi, Norihiko Obata, Satoshi Mizobuchi

**Affiliations:** Department of Anesthesiology, Kobe University Hospital, 7-5-1 Kusunoki-Cho, Chuo-ku, Kobe, Hyogo 650-0017 Japan

**Keywords:** 5-Aminolevulinic acid, Cardiac arrest, Hypotension, Bladder tumor

## Abstract

**Background:**

Although 5-aminolevulinic acid is useful for the photodynamic diagnosis of bladder tumors, it often causes severe intraoperative hypotension. We report a case of postoperative cardiac arrest in addition to severe intraoperative hypotension, probably owing to the use of 5-aminolevulinic acid.

**Case presentation:**

An 81-year-old Japanese man was scheduled to undergo transurethral resection of bladder tumor. The patient took 5-aminolevulinic acid orally 2 hours before entering the operating room. After the induction of anesthesia, his blood pressure decreased to 47/33 mmHg. The patient’s hypotension did not improve even after noradrenaline was administered. After awakening from anesthesia, the patient’s systolic blood pressure increased to approximately 100 mmHg, but approximately 5 hours after returning to the ward, cardiac arrest occurred for approximately 12 seconds.

**Conclusion:**

We experienced a case of postoperative cardiac arrest in a patient, probably owing to the use of 5-aminolevulinic acid. Although the cause of cardiac arrest is unknown, perioperative hemodynamic management must be carefully performed in patients taking 5-aminolevulinic acid.

## Background

5-Aminolevulinic acid (5-ALA) is a precursor of protoporphyrin IX (PPIX), which preferentially accumulates in cancer cells and emits strong red fluorescence upon excitation with blue light [1]. Although transurethral resection of bladder tumor (TURBT) using photodynamic diagnosis with orally administered 5-ALA has the benefit of reducing the incidence of residual tumor and tumor recurrence [2, 3], there is a known risk of severe hypotension [4]. We report a case of postoperative cardiac arrest in addition to severe intraoperative hypotension, probably owing to the use of 5-ALA.

## Case presentation

**Fig. 1:**
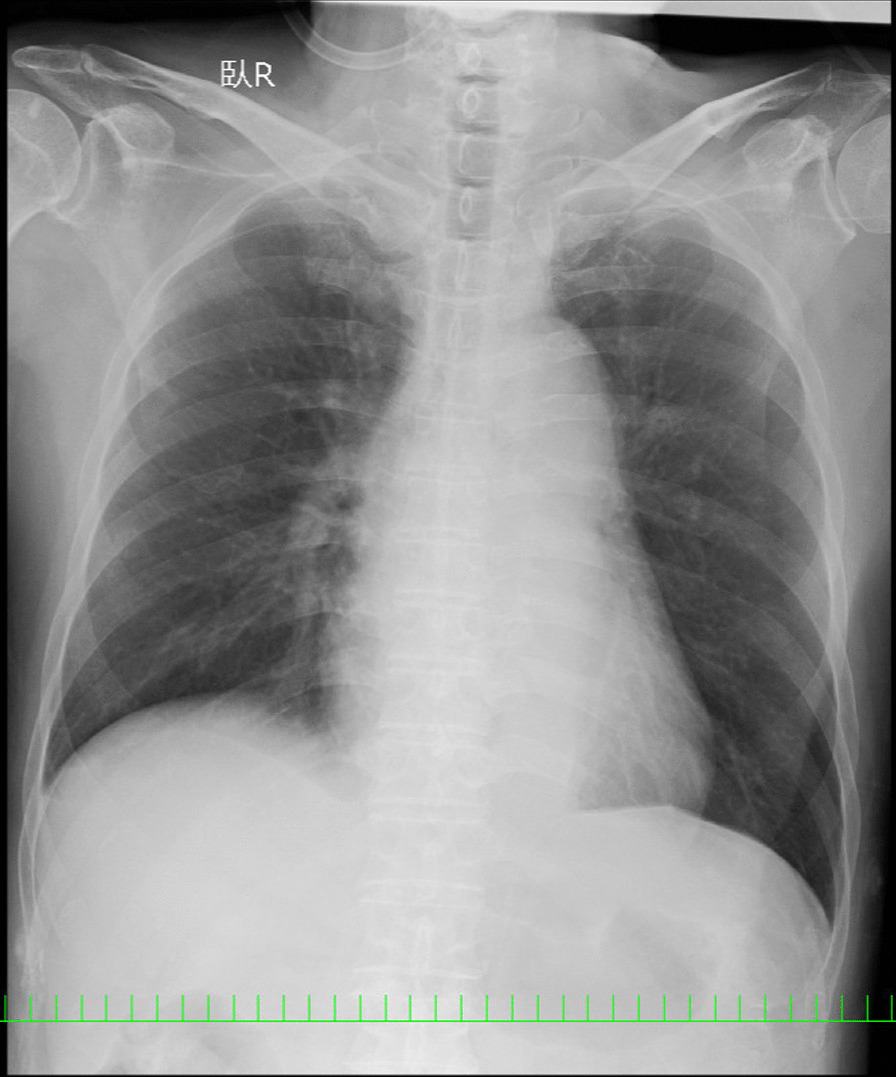
Postoperative chest X-ray

**Fig. 2:**
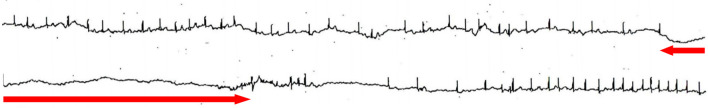
The waveform on the electrocardiogram was monitored before and after cardiac arrest. The duration of cardiac arrest is indicated by the range of the arrow. Since the waveform was 30 seconds per line, the cardiac arrest time was approximately 12 seconds

An 81-year-old Japanese man, with a weight of 58.7 kg and a height of 166 cm, was scheduled to undergo TURBT for bladder cancer. He had a history of atrial fibrillation and chronic kidney disease. He had a history of smoking 20 cigarettes per day from the age of 20 years until the age of 60 years and consumed one glass of whiskey daily. His performance status was good, and he showed no deficiencies in activities of daily living (ADL). The patient’s family and employment history were unknown. The preoperative chest X-ray was normal, but an electrocardiogram showed atrial fibrillation with a heart rate (HR) of 82 beats/minute. Blood tests taken before surgery revealed relatively high hematocrit and elevated creatinine levels (Table 1). His regular oral medications included 5 mg apixaban twice per day and 200 μg tamsulosin hydrochloride once per day, but apixaban was discontinued 2 days before surgery. He was admitted as a walk-in patient the day before surgery. A non per os (NPO) status was determined according to the American Society of Anesthesiologists (ASA) guidelines [5], and the patient stopped drinking clear liquids 2 hours before the surgery. The patient took 20 mg/kg of 5-ALA orally 2 hours before entering the operating room. At 1 hour after taking 5-ALA, his blood pressure (BP) was 118/78 mmHg. He did not feel unwell, and he walked into the operating room. An intravenous catheter was inserted into the forearm vein, and standard monitoring (electrocardiogram, noninvasive blood pressure, and pulse oximetry) was performed. The patient’s BP and HR before the induction of anesthesia were 95/67 mmHg and 110 beats/minute, respectively. After preoxygenation, general anesthesia was induced with 5 mg of remimazolam, 50 μg of fentanyl, 0.2 μg/kg/minute of remifentanil, and 40 mg of rocuronium. Anesthesia was maintained using 1 mg/kg/hour remimazolam and 0.05 μg/kg/minute remifentanil. After the induction of anesthesia, the patient’s BP decreased to 47/33 mmHg. The electrocardiogram showed atrial fibrillation, and the patient’s HR increased to approximately 135 beats/minute. A supraglottic device (SGD) was inserted for airway management. There were no findings suggestive of anaphylaxis, such as flushing or increased airway pressure. Hypotension persisted despite fluid resuscitation, head-down positioning, and repeated boluses of phenylephrine 0.1 mg (1 mg total), so a bolus of 10 μg noradrenaline (NA) was administered, followed by continuous administration of NA at 0.05 μg/kg/minute. The patient stopped taking anticoagulants before surgery, so there was a possibility that a thrombus had formed in the atrium. Considering the risk of thromboembolism, we did not perform electrical cardioversion. We administered landiolol at 3.5 μg/kg/minute, but rate control was not possible. Hypotension persisted even after NA was administered at 0.05 μg/kg/minute, so we decided to cancel the surgery and woke the patient by administering flumazenil. After awakening from anesthesia, the patient’s systolic blood pressure (sBP) increased to approximately 100 mmHg, so he was extubated. From the induction of general anesthesia to awakening, the sBP was below 80 mmHg for approximately 35 minutes. The patient was conscious and had no neurological abnormalities. The chest X-ray showed no significant changes from before the surgery (Fig. 1). After gradually discontinuing NA and observing the patient for 40 minutes, his sBP remained at at least 80 mmHg. The hospital did not have an intensive care unit (ICU), and the use of NA was not permitted in the general ward; therefore, the patient returned to the general ward after it was confirmed that his BP could be maintained without administering NA. After returning to the ward, the patient’s sBP remained at approximately 100 mmHg, but approximately 5 hours after returning to the ward (approximately 9.5 hours after the oral administration of 5-ALA), cardiac arrest and loss of consciousness occurred for approximately 12 seconds (Fig. 2). The patient recovered consciousness without the need for resuscitation measures. The patient was conscious and had no neurological abnormalities. The electrocardiogram performed immediately after the patient regained consciousness showed no significant changes from before the surgery, and arterial blood gas analysis (Table 2) revealed no obvious pH changes or electrolyte abnormalities. Blood tests conducted at the same time showed an increase in creatinine and a decrease in hematocrit (Table 1). The patient’s blood glucose level was normal at 118 mg/dL. He was taken to a hospital with an ICU and admitted to the ICU for further management and care. Echocardiography showed moderate aortic regurgitation, an ejection fraction of 59% and no asynergy. After admission to the ICU, no new medications were administered, and the patient did not experience cardiac arrest. He was discharged from the ICU the next day and was discharged from the hospital 11 days after the cardiac arrest event. The cardiologist determined that a pacemaker and new medical management were not necessary, and the patient was regularly monitored as an outpatient, but no other cardiac arrest events occurred. He underwent TURBT under general anesthesia without oral 5-ALA after 1 and 5 months but had no problems during or after surgery. The exact cause of cardiac arrest is unknown, but given these circumstances, the influence of 5-ALA cannot be ruled out.

**Table 1:** Laboratory blood tests before and after surgery

		Before surgery	After surgery
White blood cell count	(/μL)	8200	9800
Hemoglobin level	(g/dL)	16.8	12.2
Hematocrit level	(%)	47.6	34.6
Platelet count	(× 10^4^/μL)	16.5	13.9
PT-INR		1.14	1.12
APTT	(sec)	37.5	30.4
AST level	(IU/L)	18	16
ALT level	(IU/L)	18	13
Blood urea nitrogen level	(mg/dL)	25.5	24.4
Creatinine level	(mg/dL)	1.38	1.55

PT-INR, prothrombin time-international normalized ratio; APTT, activated partial thromboplastin time; AST, aspartate aminotransferase; ALT, alanine aminotransferase

**Table 2:** Arterial blood gas after cardiac arrest

pH		7.401
PaCO_2_	(mmHg)	35.2
PaO_2_	(mmHg)	86.7
HCO_3_^−^	(mmol/L)	21.4
Na^+^	(mmol/L)	136
K^+^	(mmol/L)	4.2
Cl^−^	(mmol/L)	111

## Discussion and conclusions

We experienced a case of postoperative cardiac arrest in addition to severe intraoperative hypotension, probably owing to the use of 5-ALA. Although there are many reports of severe hypotension caused by oral 5-ALA administration [4, 6–8], there have been no reports of cardiac arrest.

5-ALA is a natural biochemical precursor of heme proteins. After being absorbed, 5-ALA is metabolized through the heme biosynthetic pathway and converted into PPIX, which promotes the activation of soluble guanylate cyclase, relaxes pulmonary and systemic blood vessels, and is ultimately transformed into heme with iron by ferrochelatase in mitochondria [9, 10]. Activation of heme metabolism is thought to bring about hemodynamic changes, and in fact, higher hematocrit levels are associated with greater 5-ALA-induced perioperative blood pressure changes [10]. Blood samples taken from our patient before surgery also revealed a relatively high hematocrit level (47.6%). Furthermore, the patient had two of the three risk factors (advanced age and chronic renal failure) for severe hypotension reported by Miyakawa *et al.* [11]. Approximately 25% of 5-ALA is excreted in the urine in an unchanged form, so it is thought that patients with chronic renal failure produce a large amount of PPIX [7, 12], increasing their likelihood of developing severe hypotension. Although it cannot be ruled out that the anesthetic may have been excessive for this elderly patient, subsequent TURBT performed under general anesthesia without oral 5-ALA did not result in severe hypotension, so general anesthesia cannot be considered the sole cause of severe hypotension. The presence of atrial fibrillation may also be associated with severe hypotension. One possible method to prevent hypotension after the induction of anesthesia is to discontinue renin-angiotensin system inhibitors in patients taking them [8]. There are reports that 5-ALA increases vascular permeability [6], so excessive fasting before surgery should be avoided, and adequate fluids should be provided before and during surgery.

Patients who take 5-ALA orally often experience hypotension before surgery, and it has been reported that a preoperative (at the time of induction of anesthesia) decrease in BP may be associated with intraoperative hypotension [13]; therefore, special attention should be given to such patients. Hemodynamics should be carefully monitored during anesthesia induction, and if the response to ephedrine or phenylephrine is poor, NA, adrenaline, or vasopressin [14] should be considered. Therefore, anesthesiologists and surgeons should discuss the risks and benefits of the use of 5-ALA in patients at high risk of anesthesia, such as those with decreased cardiac function. In addition, for patients whose BP significantly decreases preoperatively, surgeons should be advised to cancel surgery.

There are no reports that oral 5-ALA administration is associated with cardiac arrest. Although the cause of this patient’s cardiac arrest was unknown, the possibility that 5-ALA and severe intraoperative hypotension played a role cannot be ruled out. Intraoperative hypotension is associated with increased 30-day major adverse cardiac or cerebrovascular events after noncardiac surgery [15], and its severity and duration are associated with postoperative organ injury [16]. Therefore, hypotension should be corrected promptly, and if BP management is still difficult, surgery should be discontinued. If a patient is under general anesthesia, their BP may recover once they awaken. Spinal anesthesia also has a risk of severe hypotension [17], so it would be better to choose an anesthesia method that is best suited to managing hypotension.

It is also necessary to differentiate 5-ALA-induced shock from drug-induced anaphylactic shock. Because the operating room is dark to prevent photosensitivity, it may be difficult to observe skin symptoms such as erythema, flushing, and urticaria. For the same reason, additional intravenous cannulation may also be difficult and should be considered prior to the induction of anesthesia.

In conclusion, we treated a patient who developed postoperative cardiac arrest in addition to severe intraoperative hypotension, probably owing to the use of 5-ALA. Although the cause of cardiac arrest is unknown, the perioperative hemodynamic management of patients taking oral 5-ALA must be carefully performed, and patients with severe intraoperative hypotension should be monitored for a long period after surgery.

## Data Availability

Not applicable.

## Abbreviations

5-ALA: 5-Aminolevulinic acid
ADL: Activities of daily living
ASA: American Society of Anesthesiologists
BP: Blood pressure
HR: Heart rate
ICU: Intensive care unit
NA: Noradrenaline
NPO: Non per os
PPIX: Protoporphyrin IX
sBP: Systolic blood pressure
SGD: Supraglottic device
TURBT: Transurethral resection of bladder tumor
